# Associations between glycolysis related metabolites and short-term mortality after ST-elevation myocardial infarction

**DOI:** 10.1186/s13098-026-02335-5

**Published:** 2026-10-28

**Authors:** Christa Meisinger, Dennis Freuer, Philip Raake, Jakob Linseisen, Timo Schmitz

**Affiliations:** 1 Epidemiology, Medical Faculty, University of Augsburg, Stenglinstraße 2, Augsburg, 86156 Germany; 2 Department of Cardiology, Respiratory Medicine and Intensive Care, University Hospital Augsburg, Augsburg, 86156 Germany

**Keywords:** Glycolysis-related metabolites, Acute myocardial infarction, Short-term mortality, Metabolic dysregulation

## Abstract

**Background:**

Prior studies found that glycolysis-related metabolic markers—particularly lactate and glucose—are associated with short-term mortality after myocardial infarction (MI). However, information on glycolysis-wide metabolite alterations and short-term post-MI mortality remains insufficient. Therefore, this study investigated the associations of glucose, lactate, pyruvate, and citrate with short-term mortality after ST-elevation myocardial infarction (STEMI).

**Methods:**

This study was based on data from 724 STEMI patients enrolled in the population-based Augsburg Myocardial Infarction Registry. Arterial EDTA blood samples were drawn at the start of the catheterization procedure and plasma concentrations of the metabolites were quantified using a high-throughput nuclear magnetic resonance (NMR) spectroscopy platform developed by Nightingale Health (Finland). Multivariable logistic regression models were used to assess the associations between glycolysis-related metabolites and 28-day mortality. P values were adjusted for multiple testing using the false discovery rate (FDR) method.

**Results:**

Overall, 677 patients survived the first 28 days after hospitalization, whereas 47 died within this period. Elevated plasma levels of glucose, lactate, pyruvate, and citrate were each significantly associated with an increased risk of short-term mortality. In the multivariable logistic regression models, the strongest association was observed for glucose (OR 2.10, 95% CI 1.53–2.88, p_adj_ ≤ 0.001) and the weakest association for pyruvate (OR 1.60, 95% CI 1.22–2.10, p_adj_ = 0.001).

**Conclusions:**

These findings indicate that metabolic disturbances, reflected by glycolysis-related metabolites, are associated with short-term mortality in STEMI patients, suggesting their potential prognostic value.

**Supplementary Information:**

The online version contains supplementary material available at https://doi.org/10.1186/s13098-026-02335-5.

## Background

Acute myocardial infarction (MI) remains a leading cause of morbidity and mortality worldwide, despite major advances in reperfusion therapy and secondary prevention [1]. However, conventional clinical and biochemical markers do not fully capture the complex metabolic disturbances that accompany ischemic myocardial injury, highlighting the need for additional biomarkers to improve risk stratification and identify patients at increased risk of adverse outcomes [2].

Cardiac ischemia induces profound alterations in myocardial energy metabolism due to a mismatch between oxygen supply and metabolic demand [3]. Under physiological conditions, the myocardium primarily depends on mitochondrial oxidative phosphorylation to meet its high energy requirements [4]. In rat hearts, during ischemia, reduced oxygen availability promotes a shift toward anaerobic glycolysis and impaired tricarboxylic acid (TCA) cycle activity [5]. These metabolic alterations may reflect severity of ischemic injury as well as systemic responses to acute metabolic stress [6].

Among glycolysis-related markers, elevated glucose concentrations have consistently been associated with adverse outcomes after MI, including in patients without known diabetes, potentially reflecting stress hyperglycemia and insulin resistance [7, 8]. Lactate has also been investigated as a marker of tissue hypoxia and hemodynamic compromise; however, its prognostic utility in acute coronary syndromes has shown inconsistent results, particularly regarding its reliability as a tissue hypoxia marker [9–11]. In addition to these established metabolic markers, intermediary metabolites such as pyruvate and citrate may provide further insight into alterations in glycolytic and mitochondrial metabolism during acute MI. Previous studies have reported significant changes in pyruvate and citrate concentrations during acute MI, with pyruvate levels decreasing and citrate levels initially increasing, although these metabolic patterns may vary according to the timing of sampling and clinical context [12–14]. However, the prognostic relevance of pyruvate and citrate in patients with acute MI remains insufficiently understood. Therefore, we investigated whether circulating concentrations of glucose, lactate, pyruvate, and citrate are associated with short-term mortality in patients with acute MI.

## Methods

### Study population

This study was based on data from the population-based Augsburg Myocardial Infarction Registry in Germany. The registry was established in 1984 within the framework of the MONICA project (Monitoring Trends and Determinants in Cardiovascular Disease). Between 1996 and 2020, it operated as the KORA (Cooperative Health Research in the Augsburg Region) Myocardial Infarction Registry, and since 2021, it has continued as the Augsburg Myocardial Infarction Registry at the University Hospital of Augsburg. The study area comprises approximately 700,000 residents and includes the city of Augsburg as well as the surrounding districts of Augsburg and Aichach-Friedberg. All consecutive patients hospitalized with acute MI in one of the seven participating hospitals were registered if they were between 25 and 74 years of age (extended to 25–84 years from 2009 onward) and resided in the study region at the time of the event. Further details regarding case ascertainment, diagnostic criteria, and quality assurance procedures have been described elsewhere [15].

The analysis was restricted to ST-elevation myocardial infarction (STEMI) patients to investigate the association within a clinically more homogeneous population characterized with an indication for urgent reperfusion therapy. This approach also reduced heterogeneity related to differences in the clinical presentation and treatment strategies between STEMI and other forms of acute coronary syndromes. For the present analysis, patients with STEMI who were admitted to the University Hospital of Augsburg between May 2009 and July 2013 were considered. Inclusion was restricted to patients who underwent transfer to the cardiac catheterization laboratory for interventional treatment following hospital admission. Patients who had undergone resuscitation before admission or had severe cardiogenic shock were not included. Consent for blood collection was obtained immediately before percutaneous coronary intervention (PCI), after the treating cardiologist had obtained consent for the PCI procedure. For patients who refused to or were unable to provide informed consent to participate no blood samples were collected and these patients were not included in the study. Overall, 724 patients with STEMI aged between 25 and 84 years were included in the analysis.

The study (original data collection) was approved by the ethics committee of the Bavarian Medical Association (Bayerische Landesärztekammer), approval number 12057. Furthermore, the collection of blood was approved by the ethics committee of the Bavarian Medical Association, approval number 09016. The study and blood collection were performed in accordance with the Declaration of Helsinki.

### Data collection

All patients were interviewed during their hospitalization by trained study nurses using a standardized questionnaire. In addition, detailed clinical information was extracted from medical records, including demographic data, cardiovascular risk factors, medical history, comorbidities, medication use, laboratory findings, and electrocardiographic results [15, 16].

Venous blood samples were collected immediately after hospital admission. Peak CK-MB was defined as the highest CK-MB value measured between hospital admission and discharge or death. CK-MB measurements during hospitalization were performed as clinically indicated and were not obtained according to a predefined standardized sampling protocol; the decision to repeat CK-MB measurements was made individually by the treating cardiologists. Consequently, the timing and frequency of CK-MB assessments varied between patients. In addition to venous blood samples, arterial EDTA blood samples were collected from the arterial access immediately before PCI and processed according to standardized protocols in the catheterization laboratory, including centrifugation, aliquoting, and storage at − 80 °C. 

### Clinical chemistry measurement

Routine blood parameters were determined from venous blood samples collected at hospital admission or during hospitalization as part of routine clinical diagnostics and treatment.

Plasma concentrations of glucose, pyruvate, lactate, and citrate were quantified in arterial EDTA blood samples using a high-throughput nuclear magnetic resonance (NMR) spectroscopy platform developed by Nightingale Health (Finland) [17, 18]. Measurements below the limit of quantification were retained in the analysis when extrapolated values were provided by Nightingale.

### Statistical analysis

Categorical variables were presented as absolute frequencies and percentages, and Chi-square tests were used for assessment of group differences. While the variable age was normally distributed and presented as mean and standard deviation (SD), all other continuous variables were non-normally distributed and thus given as median and interquartile range (IQR). For comparison of group differences, Student’s t tests (for the variable age) and Mann–Whitney U tests (for the other continuous variables) were applied.

#### Regression analysis

**Fig. 1:**
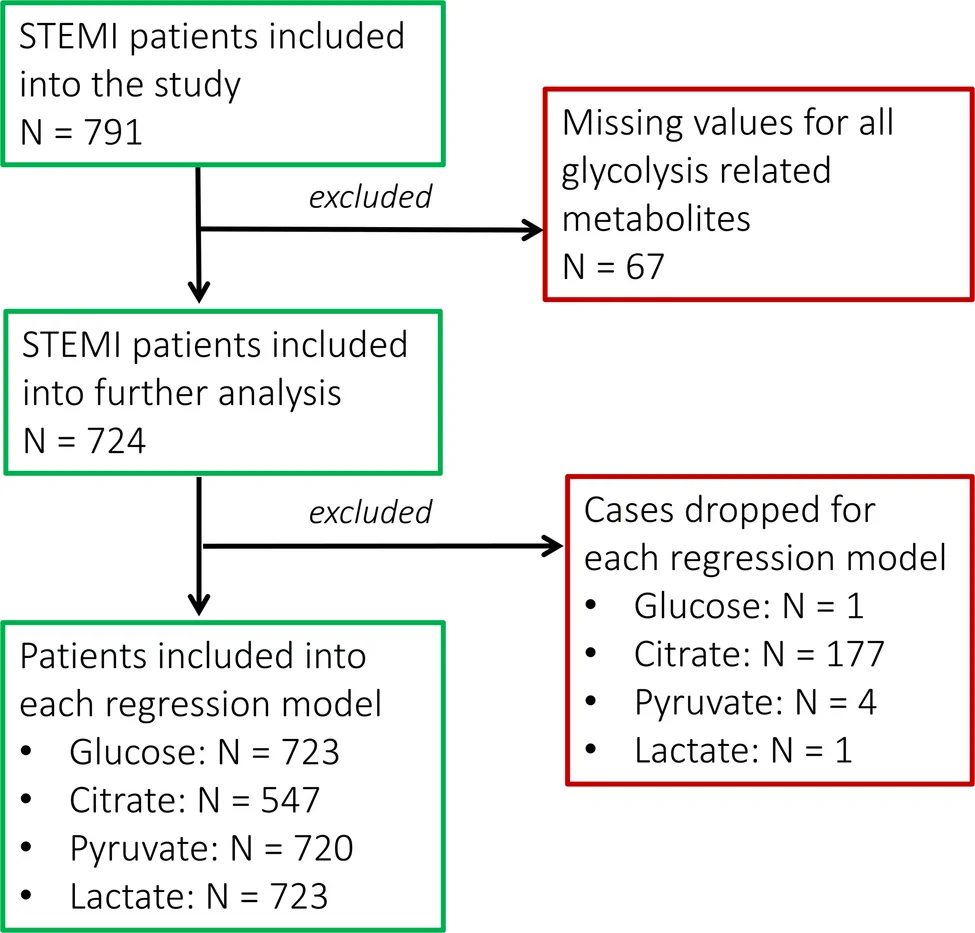
Flow chart displaying the number of cases included in each regression model

To ensure comparability across glycolysis-related metabolites, plasma concentrations of each metabolite were divided by their respective standard deviations, resulting in standardized variables with a variance of 1. Logistic regression models were used to analyze the association between glycolysis related metabolites and 28-day mortality. First, we calculated unadjusted logistic models followed by models adjusted for sex and age. Subsequently fully adjusted models were calculated. According to literature research, the models were adjusted for sex (male/female), age (in years), smoking status (current/former/never), body mass index (BMI, kg/m²), diabetes mellitus (yes/no), and renal function (normal renal function [estimated glomerular filtration rate, eGFR ≥ 60 ml/min/1.73 m²], slightly impaired renal function [eGFR 30-59.9 ml/min/1.73 m²], heavily impaired renal function [eGFR < 30 ml/min/1.73 m²]). Existing literature suggests a U-shaped association between body mass index (BMI) and mortality after MI [19], consequently we used restricted cubic splines with 3 knots (at the 10th, 50th, and 90th percentiles) for the BMI variable. Since there were substantial proportions of missing values for the variables BMI, smoking, and CK-MB levels (which was used in a subsequent sensitivity analysis), Multivariate Imputation by Chained Equations was performed using R’s mice package [20]. All missing values for covariables were imputed, the variables used for the imputation process are displayed in supplementary Table S1. While missing values for the exposure (glycolysis related metabolites) and outcome (28-day mortality) were used for the imputation process, no imputed values for the outcome and exposure were used for the regression models. Ten imputed data sets were generated. The regression models were calculated separately for each imputed data set, and the results were pooled according to Rubin´s rule [20]. The specific reasons for unsuccessful quantification of individual samples by Nightingale were not available, so that the missingness mechanism for the glycolysis related metabolites, in particular citrate, could not be determined. Therefore, no assumption of the mechanism of missingness (e.g. missing completely at random [MCAR]) was made. For the logistic regression analyses, participants with missing citrate measurements were excluded from the respective analyses (complete-case analysis regarding the exposure), see Fig. 1. The effect estimates (odds ratios and 95% confidence intervals) from the logistic regression models represent the expected odds ratio of 28-day mortality per one SD increase in the respective glycolysis-related metabolite concentration. The obtained p values were adjusted for multiple testing using the false discovery rate (FDR) method.

#### Regression assumptions and sensitivity analyses

The assumption of linearity for the metabolites of glycolysis was checked by including a quadratic term into the logistic models and checking p values of the quadratic term. Moreover, the models including the quadratic term were compared to the models including only the linear term using likelihood ratio test. Furthermore, we calculated models using monotonically transformed (square rooted and logarithmized) variables for the metabolites of glycolysis. The models were compared to the non-transformed models by calculating Nagelkerke R².

Next to the main logistic models, several additional models were calculated to evaluate robustness of the results. First, we calculated the fully adjusted model and excluded all observations with a deviation of more than three SD from the mean for the respective glycolysis related metabolites. Moreover, all observations with a Cook´s D of > 0.5 were removed from the regression models. Second, we calculated a model additionally adjusted for square rooted peak CK-MB levels to examine whether the associations are independent of infarct size. Third, Firth´s penalized logistic regression models were calculated to evaluate the potential effect of a small sample bias [21].

#### ROC analysis

In addition to the regression analyses, we performed a Receiver Operating Characteristic (ROC) analysis to evaluate whether the metabolites of glycolysis provide additional prognostic value. Therefore, we used the predicted probabilities of logistic regression models as a prediction score and calculated the area under the curve (AUC) values. The logistic models were calculated on each of the 10 imputed data sets and predicted probabilities were averaged over the 10 imputed data sets. For each metabolite of glycolysis, the performance of the logistic model was compared to a model without the corresponding metabolite (basic model including sex, age, smoking status, BMI, diabetes mellitus, and renal function). Comparison of AUC values was performed using bootstrapping. Moreover, predictive accuracy was calculated using continuous Net Reclassification Index (cNRI) and Integrated Discrimination Improvement (IDI).

All statistical analyses were performed using the statistical program R version 4.4.3. P values < 0.05 were considered statistically significant.

## Results

Due to missing values in all four glycolysis related metabolites, 67 patients were excluded from the analysis, see Fig. 1. Supplementary Table S2 compares the characteristics of included and excluded patients, showing that excluded patients were significantly older and were less likely to receive recanalization therapy. There were no significant differences regarding sex, risk factors, comorbidities, peak CK-MB, or 28-day mortality. Among the 724 STEMI patients included in the study; 677 survived the first 28 days after hospitalization, while 47 died within this period (see Table 1). The mean age of all patients was 63.4 years, with non-survivors being, on average, more than 10 years older than survivors. Approximately one quarter of the participants were women, with a significantly higher proportion among non-survivors. Survivors and non-survivors also differed significantly in several baseline characteristics, including left ventricular ejection fraction, presence of typical chest pain, and renal function. Non-survivors had significantly higher peak CK-MB values, indicating greater infarct size. In addition, all glycolysis related metabolites were significantly elevated in patients who died within the first 28 days after STEMI. Supplementary Table S3 shows the absolute numbers and frequencies of missing values in the total sample and stratified by 28-day survival status. It shows that smoking status and BMI were missing more frequently among patients who died within the first 28 days.

**Table 1:** Baseline characteristics of the STEMI patients for the total sample and stratified by 28-day mortality given as mean (SD), median (25th and 75% percentile), or absolute and relative (%) numbers

	*Total sample (N = 724)*	*28-days survived (N = 677)*	*Died within 28-days (N = 47)*	*P*-Value	*N*
Age in years	63.4 (12.6)	62.8 (12.5)	73.1 (10.2)	< 0.001	724
Female	179 (24.7)	159 (23.5)	20 (42.6)	0.006	724
First-time MI	653 (90.3)	611 (90.4)	42 (89.4)	1	723
No typical chest pain	94 (13.1)	77 (11.4)	17 (41.5)	< 0.001	716
LVEF ≥ 30%	641 (92.0)	615 (93.6)	26 (65.0)	< 0.001	697
Peak CK-MB (U/L)	155.0 (68.2; 294.0)	152.5 (67.8; 279.2)	223.0 (95.8; 408.2)	0.041	694
eGFR (ml/min/1.73 m²)	70.5 (55.8; 85.4)	72.3 (57.2; 86.1)	46.4 (36.3; 66.0)	< 0.001	721
Diabetes mellitus	198 (27.4)	184 (27.2)	14 (29.8)	0.832	723
Hypertension	519 (71.9)	483 (71.4)	36 (78.3)	0.409	722
**Smoking**				0.333	662
Never smoker	195 (29.5)	190 (29.8)	5 (20.8)		
Former smoker	178 (26.9)	173 (27.1)	5 (20.8)		
Current smoker	289 (43.7)	275 (43.1)	14 (58.3)		
**Body mass index**				0.201	690
BMI < 25 kg/m²	197 (28.6)	184 (28.0)	13 (40.6)		
BMI 25–29.9 kg/m²	312 (45.2)	302 (45.9)	10 (31.2)		
BMI ≥ 30 kg/m²	181 (26.2)	172 (26.1)	9 (28.1)		
Recanalization therapy (PTCA and/or bypass therapy)	702 (97.1)	657 (97.2)	45 (95.7)	0.904	723
**Glycolysis related metabolites measured in blood samples collected at the start of catheterization**					
Glucose (mmol/L)	7.49 (6.34; 9.32)	7.43 (6.31; 9.09)	10.57 (6.91; 13.78)	< 0.001	723
Citrate (mmol/L)	0.07 (0.06; 0.08)	0.07 (0.06; 0.08)	0.08 (0.07; 0.10)	< 0.001	547
Pyruvate (mmol/L)	0.09 (0.06; 0.13)	0.09 (0.06; 0.13)	0.10 (0.07; 0.17)	0.014	720
Lactate (mmol/L)	2.37 (1.87; 3.08)	2.31 (1.85; 3.03)	2.92 (2.19; 4.72)	< 0.001	723

LVEF: left ventricular ejection fraction; CK-MB: creatine kinase MB; eGFR: estimated glomerular filtration rate; PTCA: percutaneous transluminal coronary angioplasty;

### Regression analyses

**Fig. 2:**
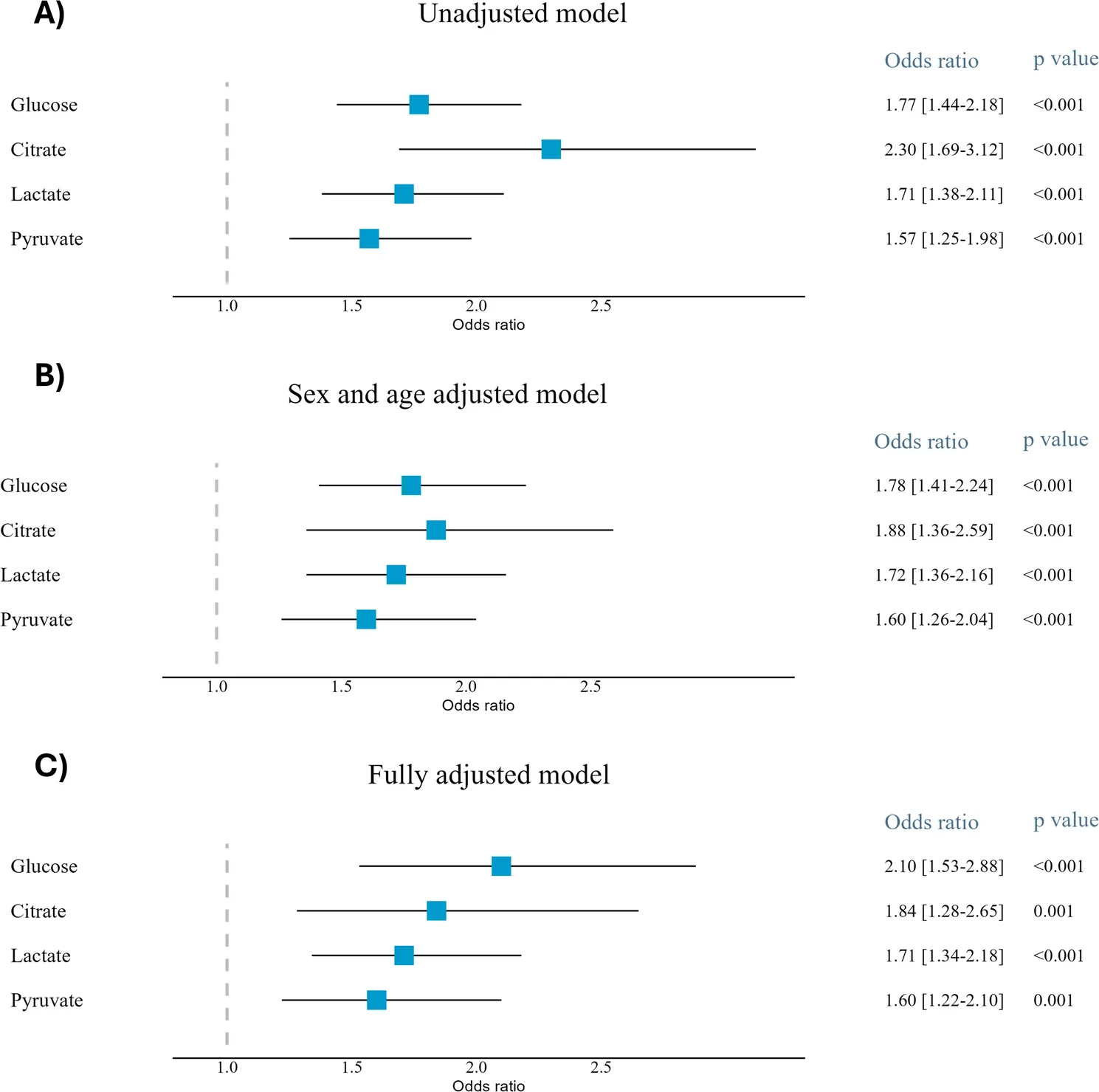
Results of the logistic regression models analyzing the association between glycolysis related metabolites (standardized concentrations) and 28-day mortality. (**A**) gives the results of the unadjusted models, (**B**) shows the models adjusted for sex and age and (**C**) gives the results of the fully adjusted models. Presented p values were FDR-adjusted, 95% confidence intervals (95% CI) were not adjusted for multiple testing

The logistic regression analyses revealed a positive association between all four examined metabolites of glycolysis and 28-day mortality in the unadjusted (Fig. 2A), sex- and age-adjusted (Fig. 2B) and fully adjusted models (Fig. 2C). The estimates varied across the different modeling approaches, indicating some sensitivity to model specification. Nevertheless, the analyses consistently suggested positive associations between glycolysis-related metabolites and 28-day mortality after STEMI. In the fully adjusted models, the strongest association was observed for glucose with an OR of 2.10 (95% CI 1.53–2.88), the weakest for pyruvate with an OR of 1.60 (95% CI 1.22–2.10). Additional information on the number of events and degrees of freedom for each model can be found in supplementary Table S4. There was no statistical indication of non-linearity for the metabolites of glycolysis and according to Nagelkerke R² the untransformed variables showed the best goodness of fit compared to the square-rooted or logarithmized variables.

Excluding influential observations and values with a deviation from the mean of more than 3 SD attenuated the effect estimates, and lactate and pyruvate were no longer significantly associated after FDR-adjustment, see supplementary Figure S1. Including square-rooted peak CK-MB levels as a covariable slightly reduced effect estimates, however all associations between metabolites of glycolysis and 28-day mortality remained significant, see supplementary Figure S2. Applying Firth´s penalized logistic regression models attenuated point estimates marginally and all associations were still highly significant, see supplementary Figure S3.

Although calculation of cNRI and IDI indicated an improved prediction accuracy, the ROC analysis revealed no notable improvement of AUC values for the metabolites, see supplementary Table S5 and supplementary Figure S4.

## Discussion

In this study, higher plasma concentrations of glucose, lactate, pyruvate, and citrate shortly before PCI were associated with increased 28-day mortality in patients with STEMI, with associations persisting across different levels of covariable adjustment and most sensitivity analyses. Glucose showed the strongest association with mortality in the fully adjusted models, whereas pyruvate showed the weakest. The addition of individual metabolites did not substantially improve AUC values. These findings suggest that glycolysis-related metabolites may reflect metabolic alterations associated with adverse outcomes after STEMI and could provide complementary prognostic information beyond established clinical predictors.

These findings are consistent with previous literature linking disturbances in glucose metabolism and impaired oxidative metabolism with adverse cardiovascular outcomes [22–24].

During cardiac ischemia, profound changes in energy metabolism occur, including a shift in substrate utilization and impairment of mitochondrial oxidative metabolism [25]. However, circulating metabolite concentrations reflect the integrated effects of production, utilization, and clearance across multiple tissues and therefore cannot be interpreted as direct measures of intracellular myocardial metabolic flux. Elevated admission glucose concentrations likely reflect a combination of stress hyperglycemia, catecholamine-driven glycogenolysis, and acute insulin resistance, all of which are known to occur during acute coronary syndromes [26].

Similarly, elevated lactate and pyruvate concentrations may reflect altered glycolytic activity, mitochondrial pyruvate utilization, or changes in systemic production and clearance [27, 28]. Importantly, circulating lactate and pyruvate concentrations alone cannot distinguish increased production from impaired utilization or clearance and therefore should not be interpreted as direct measures of glycolytic flux. Evidence supporting these mechanisms is derived primarily from studies of chronic heart failure or experimental models, and prior studies have not definitively established these mechanisms in acute human MI [27, 28]. Thus, elevated pyruvate should be regarded primarily as a marker of metabolic stress rather than a specific indicator of increased glycolysis. In contrast, elevated lactate concentrations have consistently been associated with adverse outcomes in critically ill patients [29–31]. Further studies are needed to clarify the role and prognostic significance of circulating pyruvate in acute MI. Overall, these findings are consistent with systemic metabolic alterations accompanying acute myocardial ischemia and injury, although the extent to which the observed circulating metabolite changes directly reflect myocardial metabolic alterations remains uncertain [3].

Citrate is a particularly noteworthy finding in the present study. Although citrate is classically considered a tricarboxylic acid cycle metabolite rather than a glycolytic intermediate, it is closely linked to glucose metabolism and mitochondrial metabolism [32, 33]. Citrate also contributes to the regulation of glycolysis through feedback effects on phosphofructokinase and other metabolic enzymes [34]. In addition, reviews likewise describe citrate as a feedback regulator that can restrain glycolytic enzymes and connect mitochondrial citrate production with cytosolic metabolism [33]. The observed association between higher citrate levels and increased mortality may therefore reflect broader disturbances in systemic and cardiac energy metabolism accompanying acute myocardial injury [13, 35]. Overall, these findings suggest that citrate may be a potential marker of acute cardiac stress and metabolic derangement, although its precise mechanistic significance remains unresolved. Taken together, the associations observed for glucose, lactate, pyruvate, and citrate indicate that circulating metabolites related to glucose and central carbon metabolism are associated with adverse outcomes after STEMI.

Our findings should be considered in light of several methodological strengths and limitations. Patients were consecutively recruited from the Augsburg Myocardial Infarction Registry, and blood samples were collected before PCI and processed according to standardized protocols, supporting sample quality and consistency. The extensive phenotypic characterization of patients with STEMI enabled adjustment for a broad range of clinically relevant covariables. However, the observational design limits causal interpretation, and plasma metabolite concentrations may reflect both cardiac and systemic metabolic responses rather than directly representing intracellular myocardial metabolism. Metabolites were measured only at the time of catheterization, limiting assessment of temporal changes during infarct evolution and reperfusion. CK-MB, although widely used, is only an indirect surrogate for infarct size, and variability in the timing of recanalization therapy relative to biomarker sampling may have influenced CK-MB kinetics, potentially attenuating associations. Missing data represent a further limitation. Although including the outcome in the imputation models can help account for associations between missing covariable values and mortality, it does not guarantee that the missing-at-random assumption is satisfied. The reasons for missing citrate measurements could not be established, raising the possibility of selection bias. Furthermore, excluding patients who had undergone resuscitation before admission, those with severe cardiogenic shock, and those who did not provide informed consent may have introduced additional selection bias and limited the generalizability of the findings, particularly to patients with severe clinical instability. Finally, residual confounding by unmeasured or incompletely measured factors, including comorbidities, hemodynamic instability, and treatment differences, may have influenced the observed associations. For example, information on Killip classification and the time from symptom onset to blood sampling was unavailable. In addition, although the association between lactate and mortality became statistically significant when patients with extreme lactate values were retained in the analysis, we cannot exclude the possibility that this finding was influenced by the clinical characteristics of these patients. The findings, particularly those concerning lactate, should therefore be interpreted with appropriate caution.

## Conclusion

Elevated admission concentrations of glucose, lactate, pyruvate, and citrate were associated with increased short-term mortality after STEMI. These findings suggest that disturbances in circulating metabolites related to glucose and central carbon metabolism are associated with short-term mortality after acute myocardial injury. However, as circulating metabolite concentrations do not directly reflect specific intracellular myocardial metabolic mechanisms, further studies with serial metabolite measurements, detailed metabolic phenotyping, and direct assessment of myocardial metabolism are warranted to clarify the biological basis and prognostic value of these associations.

## Supplementary Information

Below is the link to the electronic supplementary material.

Supplementary Material 1

## Data Availability

The data underlying this article cannot be shared publicly because the data are subject to national data protection laws and restrictions that were imposed by the ethics committee of the Bavarian Medical Association (“Bayerische Landesärztekammer”) to ensure data privacy of the study participants because they did not explicitly consent to the data being made publicly available. The data will be shared at reasonable requests to the corresponding author.
