# Supplementary material for: Associations between glycolysis related metabolites and short-term mortality after ST-elevation myocardial infarction
Source: Diabetol Metab Syndr. 2026 Oct 28;18:225. doi: 10.1186/s13098-026-02335-5 (PMC13718044; doi:10.1186/s13098-026-02335-5)
Supplement: Supplementary file 1 [file 13098_2026_2335_MOESM1_ESM.docx]

**Supplements**

**Table S1:** Variables used for the Multivariate Imputation by Chained Equations

| **Variable** | **Coding** |
| --- | --- |
| **Glucose** | (mmol/L) |
| **Citrate** | (mmol/L) |
| **Pyruvate** | (mmol/L) |
| **Lactate** | (mmol/L) |
| **Death within 28 days** | yes/no |
| **Age** | in years |
| **Sex** | male/female |
| **BMI** | kg/m² |
| **Nationality** | German/other |
| **First-time AMI** | yes/no |
| **Typical chest pain symptoms** | yes/no |
| **Left-ventricular ejection fraction ≥ 30%** | yes/no |
| **Diabetes** | yes/no |
| **Hypertension** | yes/no |
| **Hyperlipidemia** | yes/no |
| **eGFR** | ≥ 60 ml/min/1.73, 30-59.9 ml/min/1.73 m²,  < 30 ml/min/1.73 m² |
| **Peak CK-MB** | U/L (square rooted) |
| **PTCA** | yes/no |
| **Bypass therapy** | yes/no |

**Table S2:** Baseline characteristics of excluded vs. included STEMI patients

|  | ***Included patients (N=724)*** | ***Excluded patients (N=67)*** | ***P-Value*** | ***N*** |
| --- | --- | --- | --- | --- |
| Age in years | 63.4 (12.6) | 67.2 (11.6) | **0.015** | 791 |
| Female | 179 (24.7) | 24 (35.8) | 0.065 | 791 |
| Died within 28 days | 677 (93.5) | 63 (94.0) | 1 | 791 |
| First-time MI | 653 (90.3) | 63 (94.0) | 0.436 | 790 |
| No typical chest pain | 94 (13.1) | 9 (13.4) | 1 | 783 |
| LVEF ≥ 30% | 641 (92.0) | 61 (95.3) | 0.475 | 761 |
| Peak CK-MB (U/L) | 155.0 (68.2 - 294.0) | 171.5 (101.5 - 314.0) | 0.237 | 756 |
| eGFR (ml/min/1.73 m²) | 70.5 (55.8 - 85.4) | 66.7 (51.1 - 77.9) | 0.057 | 788 |
| Diabetes mellitus | 198 (27.4) | 13 (19.4) | 0.205 | 790 |
| Hypertension | 519 (71.9) | 49 (73.1) | 0.940 | 789 |
| **Smoking** | | | 0.714 | 721 |
| Never smoker | 195 (29.5) | 19 (32.2) |  |  |
| Former smoker | 178 (26.9) | 13 (22.0) |  |  |
| Current smoker | 289 (43.7) | 27 (45.8) |  |  |
| **Body mass index** | | | 0.201 | 690 |
| BMI < 25 kg/m² | 197 (28.6) | 19 (31.7) |  |  |
| BMI 25-29.9 kg/m² | 312 (45.2) | 33 (55.0) |  |  |
| BMI ≥ 30 kg/m² | 181 (26.2) | 8 (13.3) |  |  |
| Recanalization therapy  (PTCA and/or bypass therapy) | 702 (97.1) | 60 (89.6) | **0.004** | 790 |

**Table S3:** Number of missing values for each variable for the total sample and stratified by 28-day survival status (before imputation).

|  | Total sample (N=724) | 28-days survived (N= 677) | Died within 28-days (N=47) |
| --- | --- | --- | --- |
| Age | 0 (0) | 0 (0) | 0 (0) |
| Sex | 0 (0) | 0 (0) | 0 (0) |
| First-time MI | 1 (0.1) | 1 (0.1) | 0 (0) |
| No typical chest pain | 8 (1.1) | 2 (0.3) | 6 (12.8) |
| LVEF | 27 (3.7) | 20 (3.0) | 7 (14.9) |
| Peak CK-MB | 30 (4.1) | 29 (4.3) | 1 (2.1) |
| eGFR | 3 (0.4) | 2 (0.3) | 1 (2.1) |
| Diabetes mellitus | 1 (0.1) | 1 (0.1) | 0 (0) |
| Hypertension | 2 (0.3) | 1 (0.1) | 1 (2.1) |
| Smoking | 62 (8.6) | 39 (5.8) | 23 (48.9) |
| BMI | 34 (4.7) | 19 (2.8) | 15 (31.9) |
| Recanalization therapy | 1 (0.1) | 1 (0.1) | 0 (0) |
| Glucose | 1 (0.1) | 1 (0.1) | 0 (0) |
| Citrate | 177 (24.4) | 167 (24.7) | 10 (21.3) |
| Pyruvate | 4 (0.6) | 4 (0.6) | 0 (0) |
| Lactate | 1 (0.1) | 1 (0.1) | 0 (0) |

**Table S4:** Additional information on the main regression models.

|  | **Number of events** | **Total degrees of freedom** | **Degrees of freedom utilized** |
| --- | --- | --- | --- |
| **Glucose** | 47 | 722 | 12 |
| **Citrate** | 37 | 546 | 12 |
| **Pyruvate** | 47 | 719 | 12 |
| **Lactate** | 47 | 722 | 12 |

**Table S5:** Results of the ROC analyzes comparing the performance of logistic models for the basic score and the extended score, that is additionally including one of the four metabolites of glycolysis in the models. The predicted probabilities of the logstic models were used as score values.

|  | **AUC* (95%CI)** | **Bootstrapping**  **p value** | **cNRI**** | **cNRI**  **p value** | **IDI***** | **IDI**  **p value** |
| --- | --- | --- | --- | --- | --- | --- |
| Basic Score | 0.8455 (0.7965-0.8886) | - | - | - | - | - |
| + Glucose | 0.8628 (0.8095-0.9093) | 0.233 | 0.6353 (0.3467-0.9239) | <0.001 | 0.0761 (0.0333 - 0.1189) | <0.001 |
| Basic Score | 0.8486 (0.8486) | - | - | - | - | - |
| + Citrate | 0.8528 (0.7819-0.9092) | 0.781 | 0.4323 (0.1049 - 0.7597) | 0.010 | 0.0619 (0.0189 - 0.1049) | 0.005 |
| Basic Score | 0.8460 (0.7985-0.8928) | - | - | - | - | - |
| + Pyruvate | 0.8423 (0.7857-0.8953) | 0.752 | 0.3121 (0.0183 - 0.6059) | 0.037 | 0.0452 (0.0126 - 0.0779) | 0.007 |
| Basic Score | 0.8455 (0.796-0.8915) | - | - | - | - | - |
| + Lactate | 0.8646 (0.8103-0.9149) | 0.205 | 0.6537 (0.3625 - 0.9449) | <0.001 | 0.085 (0.0326 - 0.1373) | 0.001 |

* Area under the curve

** continous Net Reclassification Index

*** Integrated Discrimination Improvement

**Figure S1:** Results of the logistic regression models analyzing the association between glycolysis related metabolites (standardized concentrations) and 28-day mortality. Influential observations and values deviating more than three SD from the mean were excluded from the models. For glucose, 18 additional observations were excluded, 5 observations for citrate, 13 observations for pyruvate and 14 for lactate. Presented p values were FDR-adjusted, 95% confidence intervals (95% CI) were not adjusted for multiple testing.

**Figure S2:** Results of the logistic regression models analyzing the association between glycolysis related metabolites (standardized concentrations) and 28-day mortality. The models were adjusted just like the fully adjusted models and additionally for square rooted peak CKMB levels. Presented p values were FDR-adjusted, 95% confidence intervals (95% CI) were not adjusted for multiple testing.

**Figure S3:** Results of the Firth´s penalized logistic regression models analyzing the association between glycolysis related metabolites (standardized concentrations) and 28-day mortality. The models were adjusted just like the fully adjusted model. Presented p values were FDR-adjusted, 95% confidence intervals (95% CI) were not adjusted for multiple testing.

**Figure S4:** ROC curves for the basic score (green lines) and the scores supplemented by glucose (A), citrate (B), lactate (C) and pyruvate (D) respectively (blue lines).
